# The Co-Operonic PE25/PPE41 Protein Complex of *Mycobacterium tuberculosis* Elicits Increased Humoral and Cell Mediated Immune Response

**DOI:** 10.1371/journal.pone.0003586

**Published:** 2008-10-31

**Authors:** Smanla Tundup, Niteen Pathak, M. Ramanadham, Sangita Mukhopadhyay, K. J. R. Murthy, Nasreen Z. Ehtesham, Seyed E. Hasnain

**Affiliations:** 1 Laboratory of Molecular and Cellular Biology, Centre for DNA Fingerprinting and Diagnostics, Hyderabad, India; 2 Department of Biochemistry, University of Hyderabad, Hyderabad, India; 3 Immunology Unit, Mahavir Hospital, Hyderabad, India; 4 National Institute of Nutrition, Jamia Osmania, Hyderabad, India; 5 Institute of Life Sciences, HCU Campus, Hyderabad, India; 6 Jawaharlal Nehru Centre for Advanced Scientific Research, Jakkur, Bangalore, India; Emory University, United States of America

## Abstract

**Background:**

Many of the PE/PPE proteins are either surface localized or secreted outside and are thought to be a source of antigenic variation in the host. The exact role of these proteins are still elusive. We previously reported that the PPE41 protein induces high B cell response in TB patients. The PE/PPE genes are not randomly distributed in the genome but are organized as operons and the operon containing PE25 and PPE41 genes co-transcribe and their products interact with each other.

**Methodology/Principal Finding:**

We now describe the antigenic properties of the PE25, PPE41 and PE25/PPE41 protein complex coded by a single operon. The PPE41 and PE25/PPE41 protein complex induces significant (p<0.0001) B cell response in sera derived from TB patients and in mouse model as compared to the PE25 protein. Further, mice immunized with the PE25/PPE41 complex and PPE41 proteins showed significant (p<0.00001) proliferation of splenocyte as compared to the mice immunized with the PE25 protein and saline. Flow cytometric analysis showed 15–22% enhancement of CD8^+^ and CD4^+^ T cell populations when immunized with the PPE41 or PE25/PPE41 complex as compared to a marginal increase (8–10%) in the mice immunized with the PE25 protein. The PPE41 and PE25/PPE41 complex can also induce higher levels of IFN-γ, TNF-α and IL-2 cytokines.

**Conclusion:**

While this study documents the differential immunological response to the complex of PE and PPE *vis-à-vis* the individual proteins, it also highlights their potential as a candidate vaccine against tuberculosis.

## Introduction

The emergence of multi-drug resistant (MDR) and extensively drug resistant (XDR) strains has further worsened the already serious problem of TB caused by *Mycobacterium tuberculosis (M.tb)*. With no new drug in the last 40 years and the current *M. bovis* BCG vaccine providing incomplete protection in adults and varying protection, depending on infection with other organisms and geographic location [1]–[3], TB poses a still greater burden on human health globally killing a person every 16 seconds. With not much understanding about the mechanism of *M.tb*-host relation, even in the post genomic era, especially during latency, efforts are on to develop an effective and long lasting vaccine against TB. Use of subunit vaccines like ESAT-6 or Ag85 alone or together in combination with BCG or recombinant BCG has shown promise in clinical trials. Though the ESAT-6 protein is a highly immunodominant antigen eliciting strong immune response in host, recent evidences of its ability to inhibit macrophage protective functions by modulating TLR2 mediated signaling [4], target MAP kinase signaling by inhibiting ERK1/2 phosphorylation in the nucleus [5] and inhibit expression of genes under the control of NFκ-B transcription factor [6] have added to concerns about the usage of ESAT-6 as a vaccine candidate.

The host immune response against the *M.tb* infection is thought to be primarily due to cell mediated immunity, however, the importance of antibody response elicited by *M. bovis* BCG suggests that both the branches of the immune response are involved in protection against *M.tb* during early and late phase of infection [7]. The cytokines secreted by innate and adaptive immune cells have a major role in controlling *M.tb* infection by providing bactericidal activity to the host. This was evident from experiments involving mice deficient in TNF-alpha and IFN-γ cytokines which showed highest susceptibility to *M. tb* [8]–[11].

The PE/PPE proteins of *M.tb* associated with the cell wall or secreted outside by the bacterium, are well recognized by the host immune system. The genes belonging to this family, which represent 10% (167 members) of the coding capacity of the *M. tb* genome, encode proteins carrying Proline-Glutamic acid [PE) or Proline-Proline-Glutamic acid [PPE) motifs found near the N-terminus and hence the name PE/PPE [12], [13]. The PE_PGRS and PPE_MPTR are the major subfamilies of the PE and PPE families represented by their unique and repetitive domains fused to the C-terminal of the proteins carrying only the PE and PPE conserved domains, respectively. The PE/PPE family of genes are not randomly localized but are organized throughout the genome in such a way that a PE gene is always upstream to a PPE gene and is part of an operon [14]. The PPE41 (Rv2430c) and the PE25 (Rv2431c) genes are typical examples, where the proteins coded by the operon interact with each other to form a heteromer, but when alone they oligomerize [14], [15]. The PPE41 gene codes for a highly antigenic protein, which elicits strong B-cell response in TB patients [16]. It has been recently found that the PPE41 protein is secreted in human macrophages by pathogenic mycobacteria through *esx-5* locus (a homologue of ESAT-6 secretory apparatus, esx-1) [17]. Esx-5 together with esx-1 secretory system has been recently termed as type VII secretion system in mycobacteria [18]. Although PE25 gene has been shown to be essential for the secretion of the PPE41 protein and both PE25 and PPE41 proteins were identified in the culture filtrates of *M. tb* by mass spectrometric analysis [19], it is not known whether the PE25/PPE41 complex or only the PPE41 is secreted by the pathogen in the host milieu. In order to gain insight on the PE/PPE complex versus the individual PE or PPE protein, we investigated their immunogenic properties in mice as well as in TB patients. Our data, for the first time, show differential innate and adaptive immune responses against a PE/PPE protein complex and its individual components.

## Materials and Methods

### Expression and purification of recombinant proteins

The purification of the recombinant proteins was carried out as described previously [14]. The plasmids were transformed and expressed in *E. coli* BL-21 strain and the recombinant proteins were purified using Cobalt affinity chromatography. The individual proteins PE25 (rRv2430c) and PPE41 (rRv2431c) were purified by on-column refolding method using 8M-0M urea gradient [20] or by using 0.03% sarcosyl. The co-expressed proteins were eluted with 200 mM imidazole in 1× PBS from the soluble fraction by Cobalt affinity chromatography. Alternatively, the co-purified proteins were loaded on Superose 6 column for gel filtration purification of the PE25/PPE41 complex species. The sharp (∼70 kDa) peak containing both the PE25 and PPE41 proteins were collected, as described [14]. The concentration of the recombinant proteins was measured using BCA protein assay kit (Pierce). The purified proteins were treated with Polymyxin-B sepharose beads (Sigma) and the endotoxin concentration was measured using Limulus-amebocyte-lysate kit (E-TOXATE, Sigma).

### Study population

Peripheral blood was obtained from infected patients reporting to the outpatient department of the Mahavir Hospital and Research Centre, Hyderabad, India. Patients with tuberculosis (TB) were confirmed by tuberculin skin test, radiographic examination, and observation of acid-fast bacilli in sputum. All patients with confirmed diagnosis of TB were culture positive as well. Patients with fresh cases of Pulmonary TB (category I, n = 32) and those with extra-pulmonary TB (category III, n = 10) had no history of TB treatment were recruited for this study. For sera control, blood was derived from BCG vaccinated healthy volunteers (n = 10) with no evidence of TB. The Institutional Bioethics Committee approved the present study and informed consent was obtained from all the subjects.

### ELISA

ELISA was carried out to assay antibody response in TB patients and healthy controls, in 96 well plate [Corning, Costar) coated with 10 µg/ml of each recombinant protein as described [21], with slight modification. Briefly, after overnight incubation at 4°C, the plates were washed and the wells were blocked with 200 µl/well of blocking buffer. The plates were washed and further incubated with human sera (1∶200 dilution in 1×PBS) for 1 hr at 37°C. After another wash the plates were further incubated with anti-human immunoglobulin G (IgG)–horseradish peroxidase (HRP) at 1∶10,000 dilution. HRP activity was determined by using the substrate, o-phenylenediamine tetra-hydrochloride and H_2_O_2_. The reactions were terminated using 2N H_2_SO_4_ and the absorbance was measured at 492 nm in an ELISA reader.

The amount of cytokine present in the culture supernatants was quantified by commercially available two-site sandwich enzyme-linked immunosorbent assay kit (BD OptEIA™ set Mouse, IL-2, TNF-α and IFN-γ) as described [22]. Briefly, mouse splenocyte culture supernatants were harvested from parallel cultures after 48 hrs for quantification of cytokines. 96 well plates were coated with coating antibody, as suggested by the manufacturer, incubated at 4°C overnight, blocked with 2% BSA as blocking buffer and incubated for 2 hrs at 37°C. After one wash the culture supernatant was added and incubated for 2 hrs at 37°C. After another wash the detection antibody was added and incubated further for 2 hours at 37°C. The plates were once again washed and incubated with HRP conjugated secondary antibody for 1 hour at 37°C and the HRP activity was determined by using the substrate, o-phenylenediamine tetra-hydrochloride and H_2_O_2_. The reaction was terminated using 2N H_2_SO_4_ and the absorbance was measured at 492 nm in an ELISA reader.

### Isolation of splenocytes from mice

Mice were anesthetized and an incision was made from the abdominal wall, to gently remove the spleen, which was transferred aseptically to a sterile Petri-dish containing 3 ml RPMI. Spleens were crushed with frosted slides and the cells were washed with incomplete RPMI. Cells were processed further for proliferation and cytokine assays.

### Splenocyte proliferation assay

The proliferation assay with lymphocytes isolated from mice was carried out by 3[H] Thymidine incorporation method. Briefly, cells were stimulated with the recombinant proteins at different concentrations and incubated at 37°C for 2–5 days in 96 well plates. 3[H] thymidine (0.5 µci/ml) was added to each well and incubated for 24 hrs at 37°C. The cells were harvested in semi-automated harvester and the cpm was measured in a beta-scintillation counter.

### Immunization of mice

Six groups (24 mice) in two batches of female C57BL/6 mice between the age of 6–8 weeks were immunized with the PE25, PPE41 and PE25/PPE41 complex proteins (20 µg each) by giving an intraperitoneal injection using 1 ml syringe and 20 gz needle. The concentration of the PPE41 was used as a measure of the concentration of the complex protein, and this was carried out by fractionating the complex protein on SDS PAGE along with known concentration of the PPE41 or GST protein. The intensity of PPE41 protein in the PE25/PPE41 complex was calculated by comparing with different concentration of PPE41 or GST proteins and quantified using commercial software in Quantity One, Biorad. One mouse in each group received PBS injection as control.

### Intracellular staining of cytokine and extracellular staining of CD4 and CD8 receptors

Cells were stimulated with 10 µg/ml concentration of recombinant proteins and incubated for 12–36 hours. Brefeldin was added at a concentration of 10 µg/ml and incubated for 4–6 hrs. Cells were incubated with surface antibody (anti-CD4-FITC or anti-CD8-FITC) for one hr at 37°C. Cells were washed with PBS, fixed in 3% paraformaldehye, permeabilized with permeabilization buffer (0.5% saponin in PBS), washed in the same buffer followed by another wash with PBS and subsequently incubated with anti-IFN-γ-PE for FACS analysis. Extracellular staining to measure the percentage of T cell population was carried out by staining the cells with anti-CD4-FITC or anti-CD8 FITC for one hour at 37°C followed by two washes with PBS and then processed for FACS analysis.

### Statistical analysis

The statistical analysis between the groups was carried out using the online scientific calculator of GraphPad (www.graphpad.com quickcalcsttest1.cfm) to calculate mean, SEM and P values.

## Results

### The PE25/PPE41 protein complex induces a significant B cell response in TB patients

**Figure 1:**
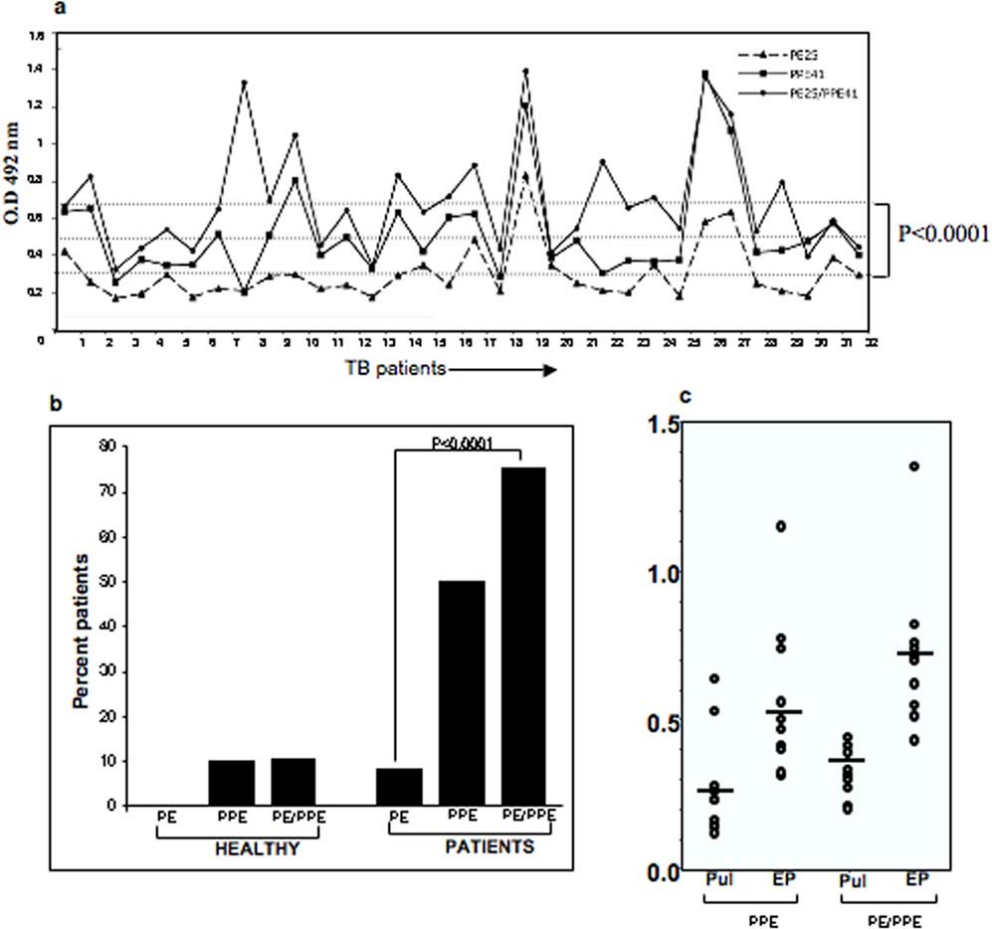
The PE25/PPE41 protein complex induces a much stronger antibody response in sera derived from TB patients. a) Sera from 32 TB patients were screened with the recombinant PE25 (dotted line with filled triangle), PPE41 (line with filled square) and PE25/PPE41 complex (line with filled circle) to score for the antibody response using ELISA. The P value (P<0.0001) is between the groups screened with the PE25 and PE25/PPE41 protein complex. The three horizontal dotted lines represent the average absorbance (O.D) of antibody response against the three proteins respectively. b) ELISA was carried out with sera from 32 TB patients and 10 healthy individuals to calculate the percentage individual reactivity to the recombinant proteins. To calculate the percentage patients showing significant antibody response against each protein, cut-off value of O. D greater than 0.55 was used. c) Sera from patients belonging to pulmonary (Pul) and extra-pulmonary (EP) categories were screened with recombinant proteins PPE41 and PE25/PPE41 protein complex using ELISA to score for the antibody response. The horizontal lines represent the average O.D of the patients of each category against each protein.

Having earlier shown that the highly immunodominant PPE41 protein discriminates TB patients from healthy individuals [16] and the genes encoding PPE41 and PE25 are co-operonic, with their products interacting with each other to form a soluble complex protein [14], we were interested to know how the host humoral system reacts to the three different species of proteins, individually and when present as a complex. Scoring for antibody response against the three species of proteins, using sera derived from TB patients (n = 32), would determine the antigenic property of the proteins during infection. Both the PPE41 and PE25/PPE41 protein complex could elicit significantly higher antibody response (P<0.0001) in TB patients as compared to the PE25 protein alone (Fig. 1a). This shows that the PE25/PPE41 protein complex also is highly immunogenic and immunodominant, similar to the PPE41 protein shown earlier by us [16]. We then checked for the number of individuals eliciting B cell response to the three recombinant protein species *viz* PE25, PPE41 and the PE25/PPE41 protein complex. As could be seen in Fig. 1b, the number of patients showing higher antibody response (O.D>0.55) to the PE25/PPE41 is more (75%) compared to the PPE41 (45%) or the PE25 protein (9%) (p<0.0001). When sera from 10 healthy individuals were used to screen the recombinant proteins, only one individual showed increased antibody titer against the PPE41 and PE25/PPE41 complex. Infection of this individual with *M.tb* could be the possible reason for the positive B cell response. The absence of any significant antibody response in sera from the other healthy individuals suggests that these proteins are mostly expressed during active infection with *M tb*. To assess the ability of the three protein species to distinguish between pulmonary (infection confined to lungs) and extra-pulmonary (infection spread in organs other than the lungs) categories, ELISA was carried out using sera from patients belonging to these categories. It could be seen that the patients belonging to extra-pulmonary category mounted higher antibody response, than those carrying pulmonary TB infection, against the PPE41 and PE25/PPE41 protein complex (Fig. 1c). While the differential response of these proteins to *M.tb* infection confined to organs other than the lungs has a possible diagnostic implication, their ability to elicit a significant higher B cell response is very apparent.

### Mice immunized with the PE25 elicit low B cell response while the PPE41 and PE25/PPE41 protein complex show increased IgG response

**Figure 2:**
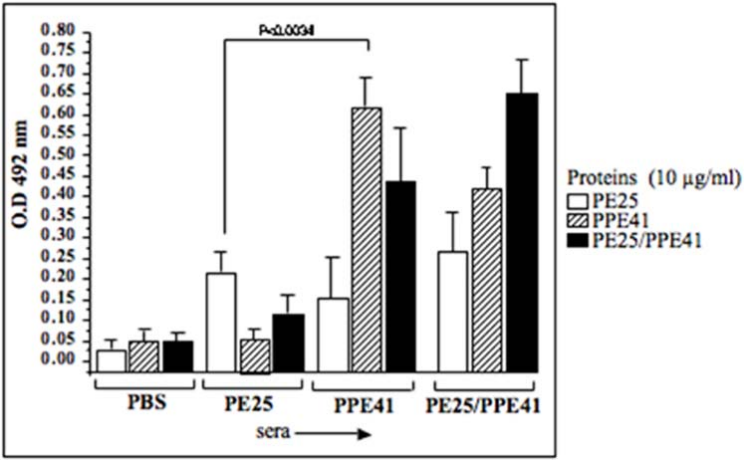
The PE25 protein induces low B cell (IgG) response whereas the PPE41 and PE25/PPE41 protein complex induce high B cell response in mice. ELISA was carried out with sera from mice immunized with the PE25, PPE41 and PE25/PPE41 recombinant proteins without any adjuvant, and screened for antibody against the respective recombinant proteins. The X-axis represents the sera from mice immunized with PBS, PE25, PPE41 and PE25/PPE41 proteins and the different bars (as indicated) represent the B cell response against each of the proteins. The p value is calculated between the groups of mice immunized with the PE25 and PPE41 proteins. The data shown represent three independent experiments carried out in triplicates.

The low antibody response against the PE25 protein in TB patients suggests that either the PE25 protein is not antigenic in nature or the antibodies raised against PE subunit of the PE25/PPE41 complex species fail to recognize recombinant PE25 protein due to incorrect conformation. This is plausible given the assumption that the proteins will be likely available in the complex form only during infection *in-vivo*. To specifically address this issue, mouse model was used to study the antigenic nature of the three recombinant protein species, as individual proteins and as a protein complex. Mice were immunized with PBS or individual PE25, PPE41 and the PE25/PPE41 protein complex without any adjuvant. Sera collected from mice after four weeks of immunization, with a booster at the second week, were used in ELISA to score for antibody response against the recombinant PE25, PPE41 and PE25/PPE41 protein complex. It can be seen that the PE25 protein generated an antibody response, which was significantly lower (p<0.0034) than the response generated by the PPE41 or that generated by the PE25/PPE41 protein complex (Fig. 2). This indicates that the PE25 protein is a weak antigen compared to the PPE41 and the PE25/PPE41 protein complex. These data are in confirmity with the observations made earlier that the PE domain of the PE_PGRS33 protein induces low antibody response in mice [23].

### The PE25/PPE41 complex protein induces high splenocyte proliferation

**Figure 3:**
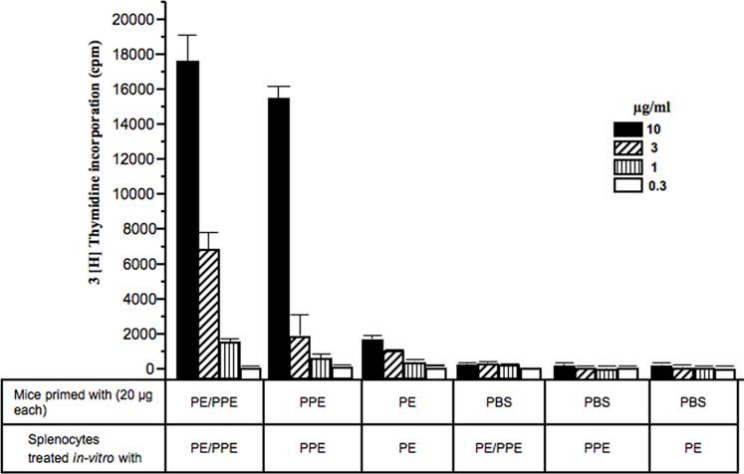
The PE25/PPE41 protein complex shows high splenocyte proliferation activity. Mice were injected with 20 µg each of the PE25, PPE41 and PE25/PPE41 proteins without any adjuvant. Splenocytes were isolated 4 weeks after the immunization with a booster dose at the second week and stimulated with corresponding proteins at different concentration *in-vitro* for 72 hours at 37°C. The proliferation activity was measured by Thymidine incorporation assay. The Different bars (as indicated) show the total thymidine (cpm) incorporated in the splenocytes isolated from mice immunized with the PE25/PPE41 complex, PPE41, PE25 and PBS (as indicated), stimulated *in-vitro* with the different concentration of the respective proteins (as indicated). The data shown represent three independent experiments carried out in triplicate.

Having observed that the PE25 protein induces low antibody response while the PPE41 and the PE25/PPE41 protein complex induces high antibody response, further studies were carried out to investigate the potential of the three recombinant protein species to elicit T cell response. Splenocytes isolated from mice immununized with PBS buffer or 20 µg of the PE25 or PPE41 or PE25/PPE41 protein complex were stimulated *in vitro* with the respective recombinant proteins. The proliferation activities of splenocytes were measured by thymidine incorporation assay. As could be seen in Fig. 3, the PE25/PPE41 complex induces higher proliferation of splenocytes as opposed to the PPE41 and PE25 protein. No proliferation was seen when splenocytes isolated from PBS immunized mice were stimulated *in-vitro* with any of the recombinant proteins using any concentration. These results once again show that the PE25 protein is indeed a weaker antigen and the high proliferative activity of the PE25/PPE41 complex could be due to the higher antigenic nature of the PPE41 species.

### The PE25/PPE41 protein complex induces IFN-γ, IL-2 and TNF-alpha cytokines

**Figure 4:**
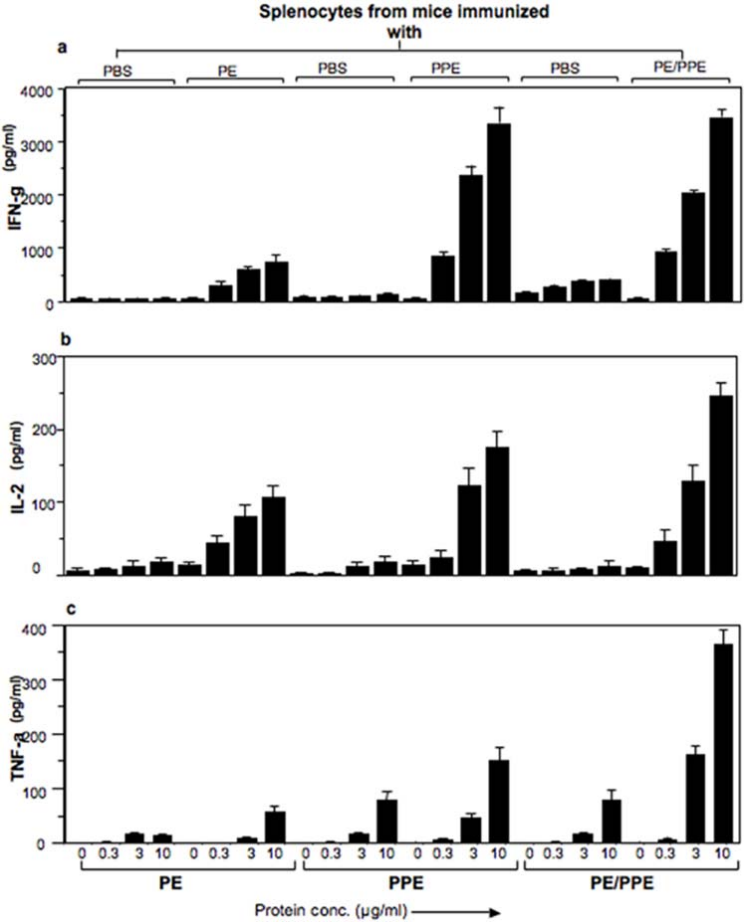
The PPE41 and the PE25/PPE41 protein complex induce strong IFN-γ, IL-2 and TNF-alpha response. Sandwich ELISA to measure cytokines, IFN-γ (a), IL-2 (b) and TNF-alpha (c) was carried out with the culture supernatants from splenocytes, isolated from mice immunized with the PE25 or PPE41 or PE25/PPE41 complex, stimulated *in-vitro* with the respective recombinant proteins at different concentrations (0 to 10 µg/ml). Data shown are representative of at least three experiments. Standard deviations were measured between the triplicate reactions.

Having shown the differential activation of mice splenocyte proliferation by the different species of the proteins *viz*, PE25, PPE41 and PE25/PPE41 protein complex; we next examined the cytokine response against these three proteins. The supernatants harvested from the respective splenocyte cultures in Fig. 3 were used to measure IFN-γ, IL-2 and TNF-alpha cytokine levels. As could be seen, when compared to mice-injected with PBS alone all the recombinant protein species could induce a significant cytokine response. However, when compared between themselves, the PPE41 and PE25/PPE41 protein complex induce higher levels of IFN-γ (Fig. 4a), IL-2 (Fig. 4b) and TNF-alpha (Fig. 4c) cytokines as compared to that induced by the PE25 protein. The induction of these cytokines was a direct function of the concentration of the recombinant protein species used to stimulate the splenocytes, with 0.3 µg/ml showing the least induction and 10 µg/ml giving the highest induction. Splenocytes isolated from mice immunized with just PBS alone do not show cytokine induction even after stimulating with the similar concentration of the PE25 or PPE41 or PE25/PPE41 protein complex. These results clearly demonstrate that the PE25/PPE41 protein complex induces the expression of cytokines typical for Th1 response in mice.

### The PPE41 and PE25/PPE41 protein complex enhance CD4^+^ and CD8^+^ T cells activity

**Figure 5:**
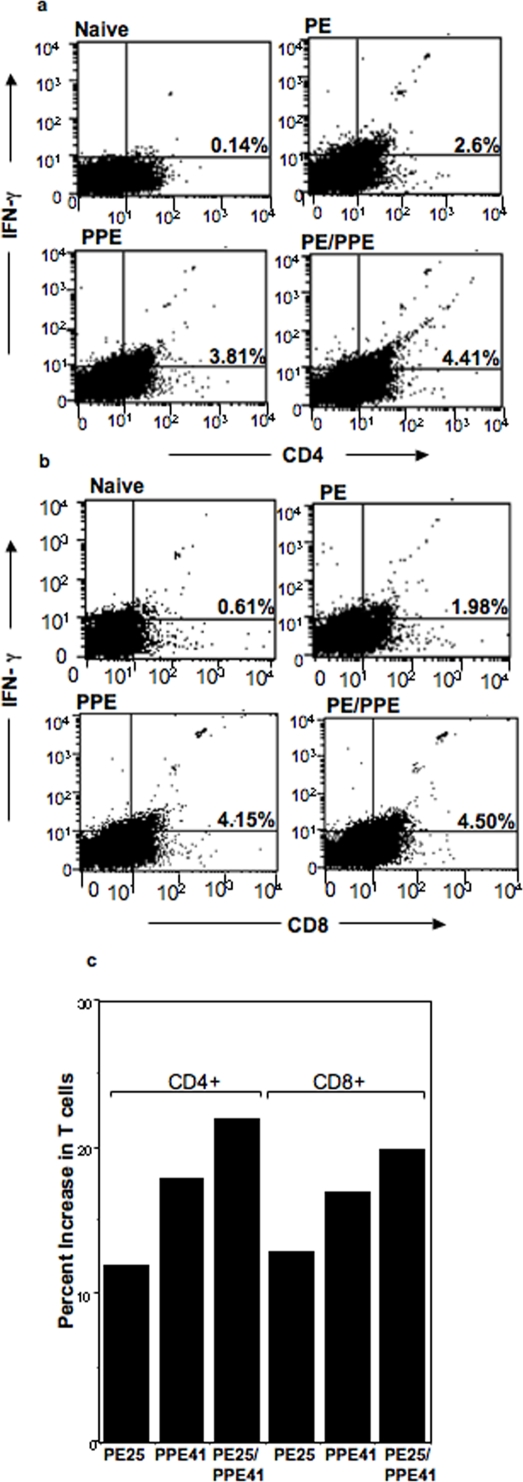
The PPE41 and PE25/PPE41 complex proteins enhance CD4^+^ and CD8^+^ T cell activity. Splenocytes from naïve mice or mice immunized with each of the recombinant proteins (PE25, PPE41 and PE25/PPE41) were stimulated with different concentration of the corresponding proteins *in-vitro*. The intracellular IFN-γ secreted by CD4^+^ (a) and CD8^+^ T cells (b) specific to the PE25, PPE41 and the PE25/PPE41 complex proteins was measured by flow cytometry and summarized as % increase in different experimental groups (c). The population of CD4^+^ and CD8^+^ T cells specific to the recombinant proteins was measured by stimulating the splenocytes with the respective proteins for 24–36 hrs. The data shown represent one of at least three similar independent experiments.

IFN-γ is extremely important for the induction of protective immunity against *M.tb*. Interestingly, we found that both the PPE41 and PE25/PPE41 complex could induce higher levels of IFN-γ. We were therefore interested in examining the type of T cells responsible for the secretion of IFN-γ. Splenocytes isolated from mice immunized with either the PE25 or PPE41 or PE25/PPE41 were stimulated *in-vitro* with the respective proteins (24 h) and stained for the CD4^+^/CD8^+^ T cells inducing IFN-γ by flow cytometry. It could be seen (Fig. 5a) that immunization with the PE25/PPE41 complex generates the maximum number of CD4^+^ (4.41%) T cells which were positive for IFN-γ staining as compared to the PPE41 (3.81%) or PE25 (2.6%) protein. Similarly, immunization with the PE25/PPE41 complex generates a larger number (4.5%) of CD8^+^ T cells which were positive for IFN-γ staining (Fig. 5b) as compared to the PPE41 (4.15%) or PE25 (1.98%) protein. No significant increase in CD4^+^ (0.14%) and CD8^+^ (0.61%) T cells were observed when cells from naïve mice or mice immunized with buffer only (data not shown) were stimulated with different species of proteins. This suggests that both CD4^+^ and CD8^+^ T cells produce higher levels of IFN-γ specific to these *M.tb* proteins. As can be seen in Fig. 5c there is an increase in the total number of CD4^+^ and CD8^+^ populations by ∼20% in response to the PE25/PPE41 complex, as compared to ∼17% for the PPE41 and ∼12% for PE25 protein. These results show that the PPE41 and PE25/PPE41 complex can activate both CD4^+^ and CD8^+^ mediated T cell response resulting in an enhanced IFN-γ response.

## Discussion

The PE/PPE family in association with *esat-6* family of genes is considered to be the “immunogenicity island” in the genome of mycobacteria based on their high immunodominant and immunopathogenic importance [24]. The PE and PPE families have expanded in the genome giving rise to a number of fused genes, belonging to the PE_PGRS and the PPE_MPTR sub-families, which are considered to be a recent evolutionary event in the genomes of *M.tb* and other pathogenic mycobacteria. About 70% of PE/PPE genes, those evolved early or in recent time, follow a pattern of genomic organization such that they are either present in clusters or fused (in the case of PE_PGRS and PPE_MPTR) (data not shown). Following upon this hypothesis and the observation that the PPE41 protein, which is a highly antigenic and immunodominant protein [16], interacts with the PE25 protein to form a soluble complex [14] and is secreted out by the bacterium in macrophages [17], we aimed to study the antigenic properties of the PE25, PPE41 and their complex PE25/PPE41 protein to gain an understanding of how host immune system responds to the three different species of the proteins derived from a single operon.

The high antibody response against the protein complex in TB patients shows that the association of the PE25 protein with the PPE41 did not affect the recognition of the PPE41 protein by the host's humoral response (Fig. 1). While it is not known whether they exist individually or as a complex in the native state, however, it is likely that in the native state this could be present as a complex and this is indeed apparent from solubility, at least in *E. coli*, of the complex. The two proteins individually exist as oligomers of 200 kDa (PE25) and 600 kDa (PPE41) where as the complex has a molecular size of approximately 70 kDa [14]. The PE25 protein has highly helical content similar to the PPE41 protein as observed by CD analysis (data not shown, [20]). The PE25 protein alone mounted very low antibody response in TB patients, which indicates that either the protein is not secreted outside to interact with the host immune system or it is not antigenic in nature. To address this question, mouse model was used to study the antigenic properties of each of the three recombinant proteins the PE25, PPE41 and PE25/PPE41 complex. The fact that even in a mouse model the PE25 protein induces very low antibody response compared to the PPE41 and PE25/PPE41 complex indicates that the PE25 is not potently antigenic. This is in agreement with earlier observation that the PE domain of the PE-PGRS33 protein does not induce significant antibody response [23]. Experiments carried out with the splenocytes isolated from mice immunized with the three recombinant proteins also showed that the PE25 protein induced low proliferation of splenocytes as compared to the PPE41 and PE25/PPE41 complex (Fig 2). The PE25 protein also showed low induction of IFN-γ and IL-2 cytokines (Fig. 3) and also low percentage of IFN-γ producing CD4^+^ and CD8^+^ T cells as analyzed by flow cytometry (Fig. 4). Therefore, it is likely that the PE protein is indeed intrinsically a weak antigen compared to the PPE41 and PE25/PPE41 complex. While different PE proteins could carry out different functions, as has been shown in the case of Rv1759c (PE_PGRS), it is the PGRS domain which elicits strong immune response [25] and, in the case of PE_PGRS33, the PGRS domain induces TNF-alpha secretion and apoptosis in macrophages [26], [27]. A recent evidence indicates that the PE domain of PE_PGRS33 and PE_PGRS11 is responsible for the translocation of the proteins to the cell wall [28]. Together all these evidences suggest that the PE domain, which helps in secretion or stabilization of the interacting proteins, is perhaps *per-se* a weak antigen and does not take part in the host-pathogen interaction. However, this may not still be considered as a general property of PE proteins in light of evidences that the PE protein induces cell death by necrosis in macrophages similar to that observed for the PPE and the PE/PPE complex (unpublished observation).

Humoral and cellular immune responses play crucial role in providing protection against *M. tb* infection. Therefore, a good subunit vaccine against *M.tb* should have the capability to induce both humoral and cellular immune response. The PPE41 and PE25/PPE41 protein complex not only induce strong humoral response but also a cellular response. The CD4^+^ and CD8^+^ T cell response form the central component of the adaptive immune response against *M.tb* infection, CD4^+^ T cells provide protection during acute phase, while the CD8^+^ T cells are critical during latency [29]–[31]. Experiments in mice showed that the PPE41 and PE25/PPE41 protein complex enhance both CD4^+^ and CD8^+^ T cell activity eliciting secretion of IFN-γ and IL-2 cytokines and also TNF-alpha. These cytokines play central role in controlling propagation of *M.tb* and thereby protecting from infection [9]–[11], [32], [33]. Not only the PE25/PPE41 protein complex is more potent in driving strong humoral and cell mediated immune responses, compared to the PPE41 protein, its soluble nature and easier steps involved in purification [14] will be advantageous in terms of its use as a vaccine candidate. The efficacy of the PE25/PPE41 in comparison to *M. bovis* BCG strain and other subunit vaccines of *M. tb* is currently being investigated.

Even though it has been shown that the PPE41 protein is expressed by *M. bovis* BCG vaccine strain too, it was confined more to the cell wall of *M. bovis* BCG as compared to the amount secreted in the culture supernatant. The pathogenic strain *M. marinum*, however, showed higher secretion of the PPE41 in the culture supernatant [17]. Although PE25 and PPE41 proteins and many other PE/PPE genes of *M. tb* shown 100% nucleotide sequence similarity with the corresponding genes of *M. bovis* BCG strain, the differential expression of these genes within the mycobacterial genus is evident, with greater expression in *M. tb* as compared to *M. bovis* BCG [34], along with key transcriptional differences between the PE/PPE genes of pathogenic mycobacteria like *M. tb* and *M. bovis* [35]. Together, these suggest that the pathogenic mycobacteria actively express the PE/PPE genes during infection and their regulation and expression correlates not only with pathogenicity but also with the specificity of the host thereby indicating that the expression or secretion of the PE/PPE genes is confined to the pathogenic mycobacteria. The lower amount of secretion of the PPE41 by *M. bovis* BCG strain could be the reason for eliciting significantly low antibody response against the PE25/PPE41 protein complex in BCG vaccinated healthy subjects (Fig. 1b). It would be, therefore, interesting to study the efficacy of not only the PE25/PPE41 protein complex alone as a vaccine, but also in combination with the existing vaccine strain, the *M. bovis* BCG.

Unlike the observation that the PE and PGRS domain of the PE-PGRS33 protein when fused fail to induce T cell response and the PE domain alone could induce T cell response [23], we show that the PE25 protein alone induces T cell response, albiet less than the PPE41 and PE25/PPE41 protein complex. That the PE25/PPE41 complex induces both a strong humoral and T cell response has a major implication in vaccine-based intervention against both early and the latent form of TB. The strong humoral response against the PPE41 and PE25/PPE41 complex proteins in extra-pulmonary patients shows that these proteins are expressed by the pathogen during infection in organs other than the lungs (Fig. 1c) further pointing to a diagnostic application of these proteins for such category of TB patients.
